# 2305. The effect of SARS-CoV-2 antibody testing on the results of admission screening

**DOI:** 10.1093/ofid/ofad500.1927

**Published:** 2023-11-27

**Authors:** Kenji Ota, Tomohiro Yamakawa, Daisuke Sasaki, Fujiko Mitsumoto-Kaseida, Norihito Kaku, Kosuke Kosai, Hiroo Hasegawa, Takahiro Takazono, Koichi Izumikawa, Hiroshi Mukae, Katsunori Yanagihara

**Affiliations:** Nagasaki University, Nagasaki, Nagasaki, Japan; Nagasaki University Hospital, Nagasaki, Nagasaki, Japan; Nagasaki University Hospital, Nagasaki, Nagasaki, Japan; Nagasaki University Graduate School of Biomedical Sciences, Nagasaki, Nagasaki, Japan; Nagasaki University Hospital, Nagasaki, Nagasaki, Japan; Nagasaki University, Nagasaki, Nagasaki, Japan; Nagasaki University, Nagasaki, Nagasaki, Japan; Nagasaki University Graduate School of Biomedical Sciences, Nagasaki, Nagasaki, Japan; Nagasaki University, Nagasaki, Nagasaki, Japan; Nagasaki University, Nagasaki, Nagasaki, Japan; Nagasaki University, Nagasaki, Nagasaki, Japan

## Abstract

**Background:**

Detection of SARS-CoV-2 in hospitalized patients is an important measure to prevent transmission to vulnerable individuals. Admission screening using nucleic acid amplification test such as PCR testing or rapid antigen testing (RAT) is proven to be beneficial in certain situations. However, the usefulness of SARS-CoV-2 antibody testing for COVID-19 screening in real-world setting is unclear.

**Methods:**

In this study, we aimed to investigate the effect of antibody testing on the results of SARS-CoV-2 screening tests. Anti-SARS-CoV-2 spike protein antibody (S-Ab) and anti-SARS-CoV-2 nucleocapsid protein antibody (N-Ab) were measured using an electrochemiluminescence immunoassay (Elecsys, Roche Diagnostics, Rotkreuz, Switzerland) in all patients who presented to the emergency department and subsequently administered to Nagasaki University Hospital. PCR testing and RAT were also performed for the detection of SARS-CoV-2. The diagnostic performance of each antibody testing to PCR and RAT was retrospectively evaluated by Fisher's exact test and odds ratio (OR).

**Results:**

A total of 2,692 patients were tested for S-Ab, N-Ab, RT-PCR, and RAT from Jan 2022 to Feb 2023. Among them, S-Ab were positive ( >0.7 U/mL) for 2,195 patients (81.5%), N-Ab ( >1.0 U/mL) 307 patients (11.4%), PCR 215 patients (7.9%), RAT 174 patients (6.5%).

For those with S-Ab > 10,000 U/mL (n=625), PCR positivity was 7.0% (*P*=0.35, OR 0.8397) and RAT 1.8% (*P*< 0.0001, OR 0.2093). For those with N-Ab positive (n=307), PCR positivity was 6.8% (*P*=0.50, OR 0.8293) and RAT 2.6% (*P*=0.0019, OR 0.3577). Comparing the group of both S-Ab > 10,000 U/mL and N-Ab positive (n=128) with that of both negative (n=2564), PCR positivity was 4.7% vs. 8.1% (Fig 1, *P*=0.1826, OR 0.5523), and RAT positivity was 1.6% vs. 6.7% (Fig 2, *P*=0.0024, OR 0.1752).

**Fig 1.**
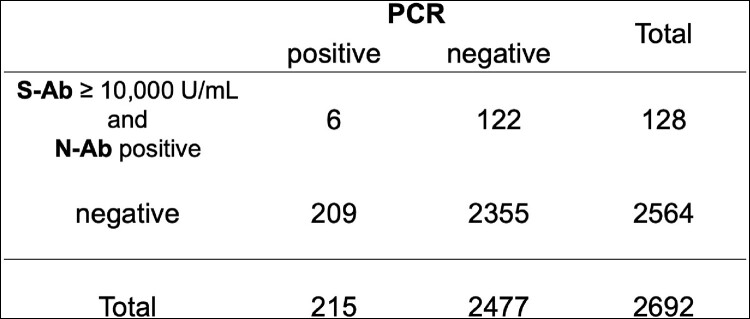
Antibody testing and PCR

**Fig 2.**
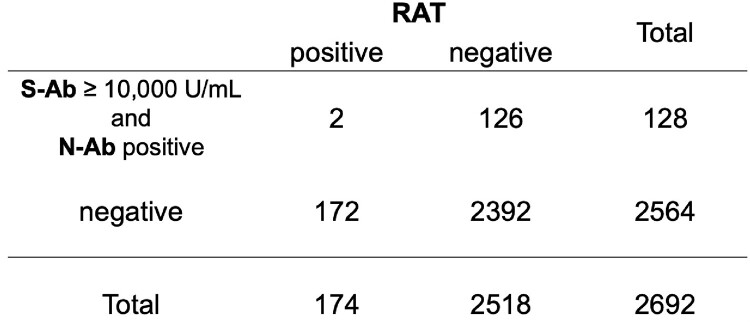
Antibody testing and RAT

**Conclusion:**

Antibody testing of S-Ab, N-Ab, or a combination of both showed performance in reducing the probability of positive PCR or RAT tests, indicating vaccine and infection-induced protective effect. However, the need for PCR and RAT tests for virus detection was suggested.

**Disclosures:**

**Kosuke Kosai, M.D., Ph.D.**, FUJIFILM Toyama Chemical Co., Ltd.: Commissioned research|KYORIN Pharmaceutical Co.,Ltd.: Commissioned research **Koichi Izumikawa, M.D., Ph.D.**, Asahi Kasei Pharma Corporation: Grant/Research Support|Asahi Kasei Pharma Corporation: Honoraria|Astellas Pharma Inc.: Honoraria|DAIICHI SANKYO COMPANY, LIMITED: Grant/Research Support|DAIICHI SANKYO COMPANY, LIMITED: Honoraria|KYORIN Pharmaceutical Co.,Ltd.: Honoraria|Merck & Co., Inc.: Honoraria|Pfizer Japan Inc.: Honoraria|Shionogi & Co., Ltd.: Grant/Research Support|Shionogi & Co., Ltd.: Honoraria|Sumitomo Pharma Co., Ltd.: Grant/Research Support|Sumitomo Pharma Co., Ltd.: Honoraria|TAIHO PHARMACEUTICAL CO., LTD.: Grant/Research Support **Katsunori Yanagihara, MD, PhD**, FUJIFILM Toyama Chemical Co., Ltd.: Commissioned research|KYORIN Pharmaceutical Co.,Ltd.: Commissioned research

